# Practical Aids in Medical and Surgical Diagnosis

**Published:** 1910-09

**Authors:** Lewis M. Gaines

**Affiliations:** Atlanta, Ga.; Professor of Physiology, Atlanta School of Medicine, Assoc. Medical Director, Peachtree Heights Sanatorium; Physician to College Hospital of A. S. of M.


### *PRACTICAL AIDS IN MEDICAL AND SURGICAL DIAGNOSIS.

By Lewis M. Gaines, A. B., M. D., Atlanta, Ga.

*Professor of Physiology, Atlanta School of Medicine, Assoc. Medical Director, Peachtree Heights Sanatorium; Physician to College Hospital of A. S. of M.*

### II.

### THE PRACTICAL VALUE OF URINE EXAMINATIONS.

Few secretions or excretions of the human organisms throw more light upon the nature of disease than the urine, which is a fluid of the most remarkable complexity. Few diseased conditions of any gravity arise in which it is not advantageous at more or less frequent intervals to subject this excretion to a careful examination.

Volumes have been written upon the subject of urine examinations, and in the space of a brief article it would be folly to attempt a full discussion of so large a subject. I desire, however, to call attention to certain points in the examination of the urine, which I have been led to believe are neglected, and to inquire, also, what particular examinations of the urine are of real practical value, and in what conditions. And finally, I desire to call attention to the clinical interpretations which should and should not be placed upon various findings in the urine.

The detection of albumin in the urine is usually the main object the physician has in mind when he begins an examination, and yet it is remarkable how often it remains undetected. After examining for life insurance companies a great many hundred specimens, which had been previously examined oft-times by various physicians over different parts of the country, frequently albumen in small traces has appeared. I have concluded that the trouble is with the method employed.

*This paper is the second of a series on Practical Aids in Medical and Surgical Diagnosis. The last paper was on the Practical Value of Blood Examinations. A reprint will be sent by the author if requested.

After experimenting with many methods, I have adopted the following: The urine should always be filtered and perfectly clear, as otherwise the test is useless. If bacteria are present in the urine rendering it turbid, simple filtration will not clarify it, as bacteria readily passes through filter paper. In such a case the urine may be clarified by adding to it a few drops of ammonium hydroxide which precipitates the earthy phosphates, the latter carrying the bacteria down in the precipitate which may then be readily filtered. After filtration the urine is rendered faintly acid with 10% nitric acid, and is then ready for the albumin test. A small, clean test-tube is filled two-thirds full of urine of acid reaction, and to this urine is added one-sixth its volume of a saturated solution of sodium chloride. The upper portion of the tube is now boiled, and then from a pipette is added three drops of 10% nitric acid. The urine is again brought to the boil and three more drops added. The urine is now ready for inspection, but the mode of inspection is very important. The urine should be viewed in good daylight (not an artificial light), and the test-tube should be held to the light, but against a black back ground. I always use a small board covered with black velvet, holding the lower portion of the test-tube in the right hand, and the black board immediately behind the tube, standing the while in front of the window. Should albumin be present it is seen as either a faint bluish white haze in the case of the faintest possible trace, or a white cloud in the case of a trace, or a heavy white precipitate if much albumen be present. Of course the value of the careful technic is in discovering minute traces of albumin, as any of the tests even perhaps carelessly employed would discover a large amount. In diagnosing chronic interstitial nephritis, and in life insurance work, it is the faintest possible trace which we want to demonstrate, if present.

The object in putting sodium chloride in the urine is to prevent the precipitation of nucleoalbumin which is without pathological significance as far as known. Acids, particularly acetic acid, do not cause a precipitation of nucleoalbumin in the presence of an excess of sodium chloride, whereas the precipitation of serum albumin is not interfered with.

A second examination of the urine of great importance, and yet often neglected, is the estimation of the total amount of urea being excreted. This assumes its greatest importance in examining the urine of pregnant women. We are, of course, on the lookout for eclampsia. Too frequently the only examination made is for albumin. Many cases of eclampsia are never albuminuric. Frequently, too, if the urea output is examined for, only the percentage amount is estimated. This is useless, as no clue is thereby given to the absolute amount. The only way to arrive at the real trustworthy information is to obtain the twenty-four-hour quantity passed, and then by examining a specimen from this entire quantity calculate the output for twenty-four hours.

A third examination of the utmost importance is the microscopic examination of the urine. It has been recently demonstrated that a certain portion of cases of nephritis never have a demonstrable amount of albumin present, and yet the condition may be suspected from the microscopic examination. The most frequent findings in such cases are casts very few in number, and usually of the granular or hyaline variety, and red blood corpuscles also usually rather scanty. Such findings, together with a low specific gravity, particularly if polyuria be present, is strong presumptive evidence of interstitial change in the kidneys.

What particular examinations of the urine are of the greatest practical value? In all cases of serious or puzzling sickness the urine should be subjected to what must be called a routine examination, which should include the following procedures: Examination of color, transparency, reaction, specific gravity; tests for albumin and sugar, microscopic examination of the centrifugalized sediment, for the presence of casts, pus cells, red blood cells, crystals, epithelium, mucus and other more rarely present abnormalities.

Why are the above estimations and tests of value? The color of urine often affords a clue as to some abnormal content. For example, bile in urine often gives it a peculiar tint. On shaking such a urine it foams very readily, and the foam has a golden yellow tint. By this is suggested the wisdom of Gmelin's test for bile. Such a urine recently came under my observation in a routine life insurance examination. The company was very glad to know the applicant had bile in the urine. Blood or blood coloring matter, or chyle, may be suspected from the color. The urine of diabetes is usually a peculiar very pale shade, and of course of high specific gravity as a rule. The transparency of urine is of value when taken in connection with the reaction. A recent experience will illustrate: A man 57 years of age of magnificent physique presented himself for examination for life insurance. The examination concluded with the examination of the urine. Up to this time he had passed a perfect examination, while he had given no suspicious history of past illness. He passed the urine in my presence, and I noticed it was quite turbid and of acid reaction. Such an occurrence usually means urates which dissolve on heating, and are of no moment. But this turbidity persisted after the urine was boiled. All that remained was to scrutinize the sediment under the microscope, and there was revealed a very large amount of pus. Close questioning in view of the finding brought out the fact that the man had suffered a number of attacks of kidney colic and had passed gravel. There was doubtless present a chronic suppurative pyelitis, and of course the risk was a bad one from the Insurance Company's point of view.

The test for the specific gravity is of considerable importance. A high or low specific gravity may mean disease or health. It must be considered in connection with other findings. In the urine of chronic interstitial nephritis, its significance is most marked, for here it is one of the few abnormalities constantly present. Where specific gravity is constantly 1010 or lower in spite of regulation of fluid intake, and stimulating diuretics, interstitial changes may be suspected.

For many years there has been sought for a reliable test whereby the functional activity or capability of the kidneys could be estimated. Many tests have been proposed, but none have proven trustworthy until a recent one very lately worked out by Rowntree and Geraghty of Baltimore. This test holds out excellent promise of proving of the greatest value. In cases about to be subjected to operation it is frequently of the utmost importance to know whether or not the kidneys possess a functional capacity sufficient to avert the dangers of uraemia or anuria. Over 200 functional tests have been performed by Rowntree and Geraghty with a substance known as Phenolsulphonephthalein. In normal cases this substance appears in the urine in from five to ten minutes after being administered, and 40 to 60 per cent. of the 6 gm. dose is recovered in the first hour. From 15 to 20 per cent. of the drug is recovered the second hour, making in all 60 to 85 per cent. recovered. In cases where the kidney function is impaired the drug appears only after a long interval, and in very small amount. Thus in two cases of chronic interstitial nephritis less than 1 per cent. of the drug was recovered in the course of an hour. Both patients died of uraemia within two months. Furthermore, where such a low grade of functional activity is demonstrated much can be done to stimulate activity and bring the kidney up to the point where the patient can pass safely through operation, or to benefit the condition in cases where no operation is contemplated. It is of value therefore to make frequent tests to ascertain the condition from time to time.

The careful and accurate study of the urine from many standpoints is of great value in successfully diagnosing a multitude of disorders. This paper has only touched on a few points of interest; there are many others of equal importance. The study of the urine is more widespread than ever, but its importance should be more universally recognized, and each physician should develop his own technic out of the best methods and carefully practice them. If this is not feasable, he should have his specimens of urine examined for him.

823 Candler Building.

(The next paper of this series will be on the practical value of sputum examinations.)

